# Radiologic Evaluation of Subcutaneous Fat Necrosis Complicated by an Abscess in a Newborn: A Case Report

**DOI:** 10.7759/cureus.87247

**Published:** 2025-07-03

**Authors:** Yazan E Al-Kharabsheh, Edvin Isufi, James Brown, Cecilia Gadaga, Maaz Ghouri

**Affiliations:** 1 Department of Radiology, University of Missouri School of Medicine, Columbia, USA; 2 Department of Pediatrics, University of Missouri School of Medicine, Columbia, USA

**Keywords:** hypercalcemia, newborn, paediatric radiology, subcutaneous abscess, subcutaneous fat necrosis, ultrasound imaging

## Abstract

Subcutaneous fat necrosis of the newborn (SCFN) is a condition that may affect neonates within the first weeks of life. SCFN lesions often present as firm, well-defined nodules or plaques under the skin, accompanied by redness or discoloration. These lesions typically present on areas such as the trunk, limbs, or cheeks. SCFN generally resolves on its own but may be associated with metabolic or hematologic complications. This report describes the sonographic findings in a unique case of a seven-day-old female neonate with SCFN complicated by both hypercalcemia and a subcutaneous abscess. The report highlights the importance of considering infection in atypical presentations of SCFN and the utility of ultrasound imaging in characterizing the lesion.

## Introduction

Subcutaneous fat necrosis of the newborn (SCFN) is a rare, self-limited form of panniculitis that affects neonates within the first few weeks of life [1,2]. SCFN lesions typically present as erythematous or violaceous, indurated, well-demarcated subcutaneous nodules or plaques, and may appear on the trunk, proximal extremities, or cheeks [1]. Although the exact etiology remains unclear, SCFN has been associated with perinatal stressors, such as hypoxia and therapeutic hypothermia, as well as gestational complications, including maternal diabetes, maternal hypertension, hypothyroidism, or pre-eclampsia [1,2]. It occurs more frequently in term neonates [1].

SCFN generally resolves without intervention; however, it may be accompanied by serious complications. One potentially life-threatening complication reported in many cases is hypercalcemia [1]. Other rare complications include hypertriglyceridemia, anemia, hypoglycemia, and thrombocytopenia [1,3]. Additional complications that may occur in the area of the lesion include ulceration, infection, or scarring [4].

SCFN is primarily a clinical diagnosis, though biopsy remains the gold standard for confirmation [1]. Ultrasound imaging can serve as a supportive modality; however, findings may be difficult to distinguish from those of cellulitis or abscess [5,6]. In this paper, we describe an atypical presentation of a subcutaneous abscess with underlying SCFN in a seven-day-old neonate with hypercalcemia.

## Case presentation

We report the case of a female neonate born at 40 weeks and 6 days of gestation via home vaginal delivery. The delivery was attended by the maternal grandmother, who served as an informal midwife. The neonate's birth weight was 3,486 grams, and the length was 20 inches. Apgar scores were 9 and 10 at one and five minutes, respectively. According to the family, the postnatal course was otherwise unremarkable. The pregnancy was characterized by limited prenatal care, and the maternal Group B Streptococcus (GBS) status was unknown. No perinatal antibiotic prophylaxis was administered. The patient did not receive the hepatitis B vaccine, intramuscular vitamin K, or ophthalmic erythromycin. The patient had not been evaluated by a pediatrician prior to their presentation to the emergency department (ED).

The patient presented on day of life (DOL) seven to the pediatric ED with concern for respiratory distress and localized erythema and swelling of the left anterior chest wall. Additional symptoms included irritability, decreased oral intake, and a reported fever. The erythema had initially been noted on DOL six and had progressively enlarged, becoming increasingly swollen and tender.

**Figure 1:**
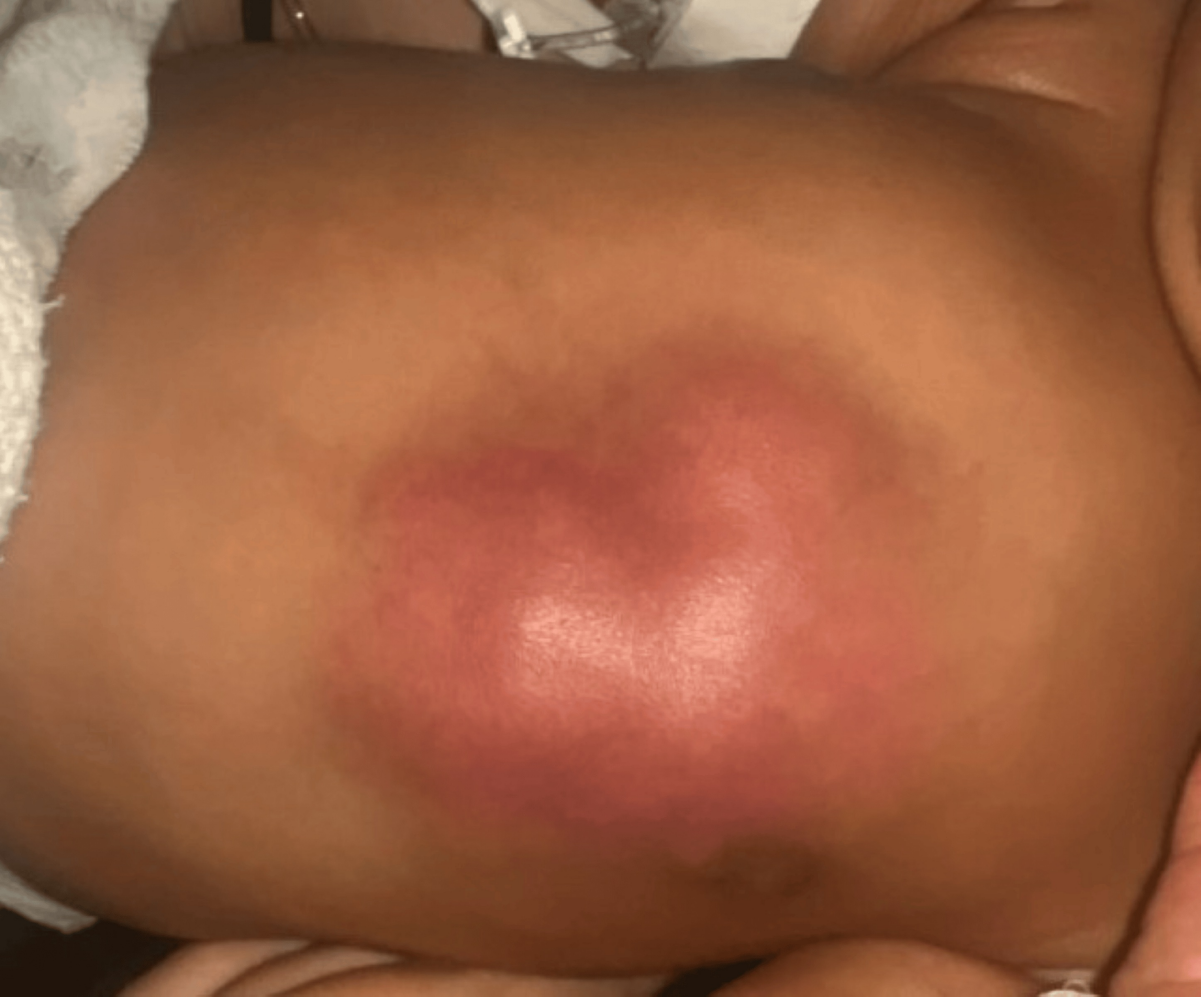
A nodular, erythematous to red-yellow, irregular, well-demarcated mass measuring approximately 4×5 cm

On physical examination, the neonate was febrile and tachycardic. Examination of the left chest exhibited a tender, nodular, erythematous to a red-yellow, irregular, well-demarcated mass measuring approximately 4 × 5 cm, without expressible fluid (Figure 1). An ED ultrasound (US) revealed findings concerning for cellulitis. Initial laboratory evaluation showed white blood cells at 19.85 x10⁹/L, hemoglobin at 17.5 g/dL, hematocrit at 50.2%, platelets at 246 x10⁹/L, aspartate transaminase at 25 U/L, alanine transaminase at 18 U/L, elevated procalcitonin at 0.68 ng/mL (normal, <=0.05), elevated serum calcium at 10.7 mg/dL (normal, 8.3-10.6), and serum lactate at 1.9 mmol/L. Urinalysis and blood cultures were normal. Empiric vancomycin was initiated due to a positive nasal swab testing for methicillin-resistant Staphylococcus aureus (MRSA) in addition to the patient’s respiratory symptoms.

The patient was admitted to the pediatric ward with a working diagnosis of cellulitis. Due to clinical concern for MRSA skin and soft tissue infection, empiric intravenous vancomycin was continued. On DOL eight, pediatric surgery was consulted. Examination of the lesion revealed a small area of induration without fluctuance or purulent drainage, with no indication for surgical intervention at that time.

**Figure 2:**
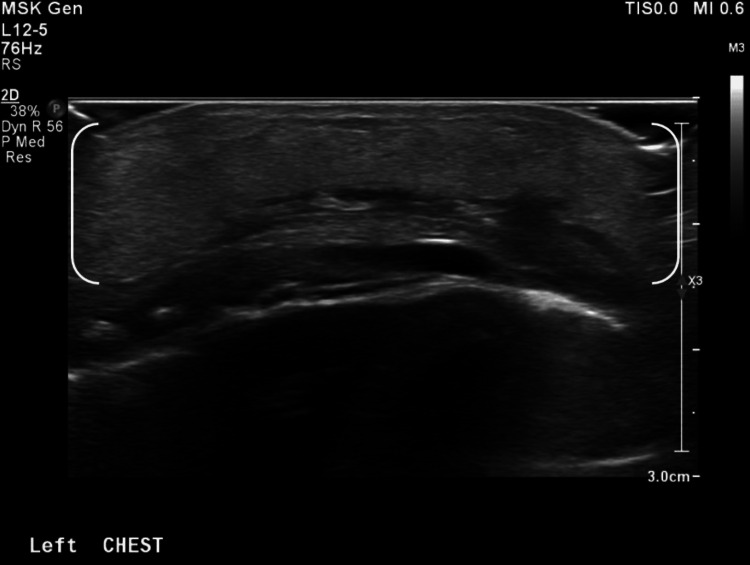
Ultrasound of the left chest wall demonstrating diffuse echogenic widening of the subcutaneous tissues with mild hyperemia

**Figure 3:**
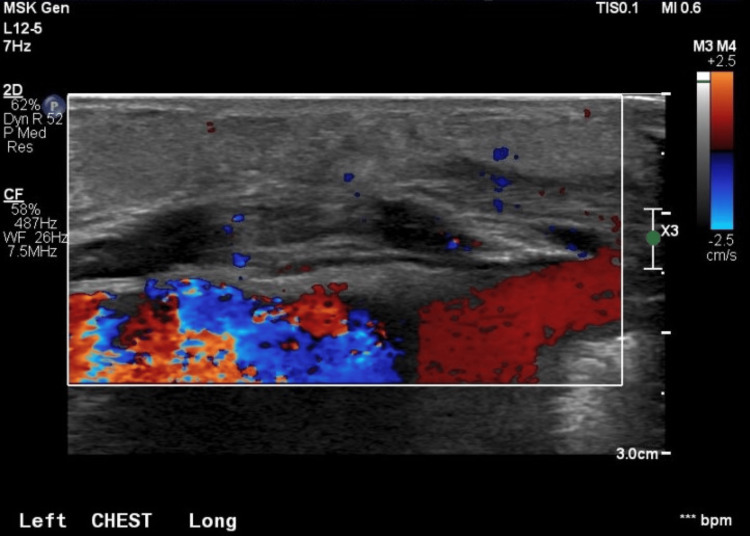
Ultrasound imaging showing a lack of vascular component increase in subcutaneous tissue

On DOL 9 and 10, the area of induration and fluctuance increased, while the overlying erythema remained unchanged. Repeat ultrasound of the left chest wall demonstrated diffuse echogenic widening of the subcutaneous tissues with mild hyperemia, findings consistent with inflammation or cellulitis (Figure 2). No solid or vascular components, calcifications, focal abscess, or free fluid were identified (Figure 3).

**Figure 4:**
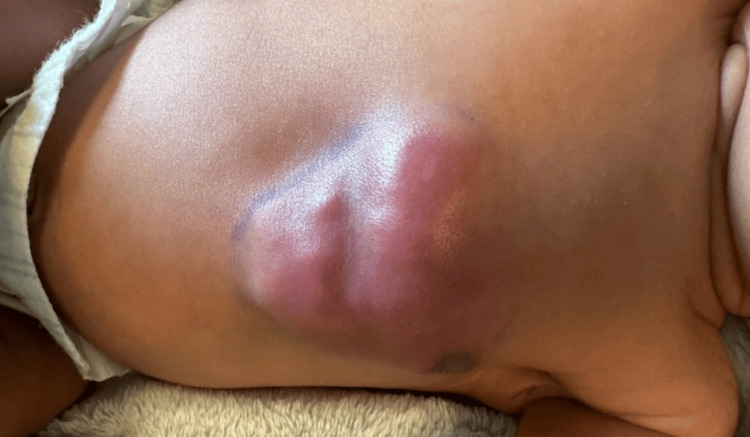
Increased fluctuance and swelling on DOL 11 DOL: day of life

**Figure 5:**
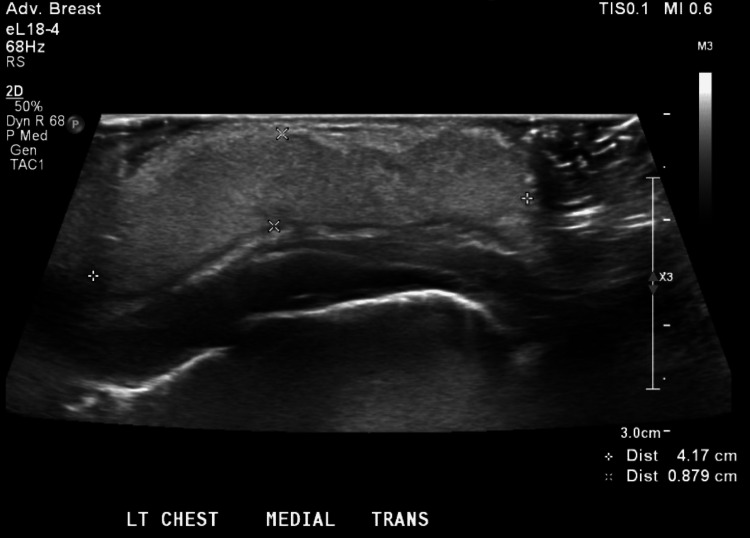
Diffuse homogeneously echogenic widening of the subcutaneous tissues measuring 4.2 cm in the transverse dimension by 0.9 cm in the anteroposterior dimension

**Figure 6:**
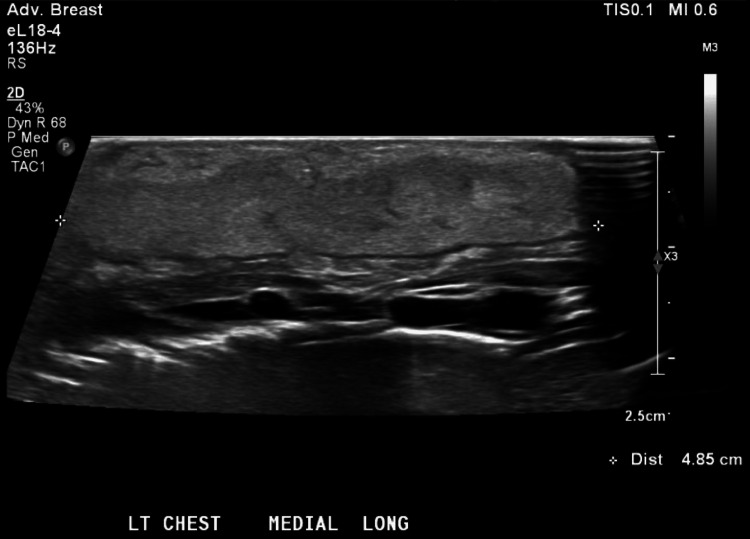
Diffuse homogeneously echogenic widening of the subcutaneous tissues measuring 4.9 cm in the longitudinal dimension

On DOL 11, the erythema showed slight improvement, but fluctuance and swelling continued to evolve (Figure 4). Serum calcium and ionized calcium levels on DOL 12 increased to 11.5 mg/dL and 1.32 mmol/L (normal, 1.12-1.30), respectively. A follow-up ultrasound demonstrated continued diffuse echogenic thickening of the subcutaneous tissues (4.9 × 4.2 × 0.9 cm) (Figures 5, 6). The imaging findings were considered most consistent with subcutaneous fat necrosis of the newborn; however, cellulitis remained within the differential diagnosis, as it is similar in appearance on US imaging.

**Figure 7:**
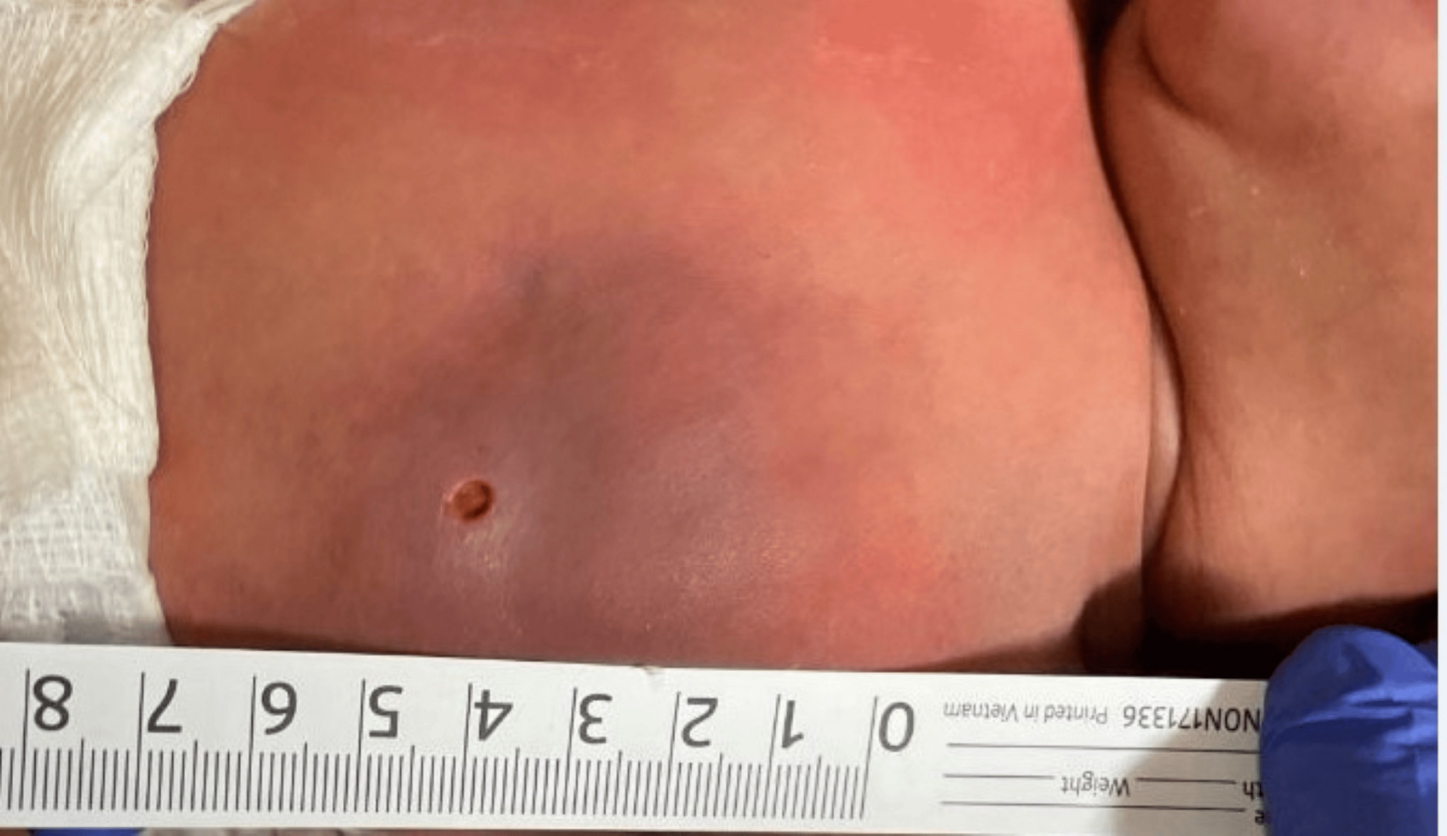
Incision and drainage of the lesion

Following a dermatology consultation, incision and drainage of the lesion were performed (Figure 7); pressurized purulent material was released, confirming the presence of a deep abscess. Approximately 7 mL of seropurulent fluid was expressed, and no solid mass was identified. The collected material was sent for aerobic and anaerobic cultures, which later confirmed moderate growth of MRSA.

The patient was discharged on DOL 13 with a diagnosis of a left chest wall abscess due to MRSA with a component of SCFN and was transitioned from vancomycin to oral clindamycin. On discharge, ionized calcium was 1.29 mmol/L. On DOL 14, serum calcium remained elevated at 11.6 mg/dL, with an ionized calcium level of 1.16 mmol/L. Given concern for SCFN and the ongoing elevation in serum calcium levels, it was recommended that the blood work be repeated at one week from discharge.

## Discussion

SCFN is a rare, self-limited form of panniculitis that typically affects neonates within the first few weeks of life [1,2]. Lesions usually present as erythematous or violaceous, indurated, well-demarcated subcutaneous nodules or plaques, commonly appearing on the trunk, proximal extremities, or cheeks [1,2]. SCFN occurs more frequently in term neonates, though several cases have also been reported in preterm neonates [1].

While the exact etiology of SCFN remains unclear, it has been associated with perinatal stressors such as hypoxia and mechanical pressure [1]. Therapeutic hypothermia, often used for neonates with perinatal asphyxia, has also been implicated in the development of SCFN [1,2]. Additional risk factors include maternal conditions such as diabetes, hypertension, hypothyroidism, or preeclampsia [1,2].

Although SCFN generally has a favorable prognosis and often resolves without intervention, it may be accompanied by significant complications. One of the most serious, common, and potentially life-threatening complications is hypercalcemia. The mechanism of hypercalcemia is thought to involve macrophages in the SCFN lesions that produce 1,25-dihydroxyvitamin D [1]. Given the high prevalence of this complication, it is recommended that all infants diagnosed with SCFN undergo periodic monitoring of serum calcium levels and be screened for nephrocalcinosis until calcium levels normalize [1].

Other reported complications, though less common and typically mild, include hypertriglyceridemia, anemia, hypoglycemia, and thrombocytopenia [1]. Local complications may involve ulceration and scarring [4]. Infection is considered extremely rare, but it has been described in isolated case reports [4,7]. SCFN may mimic skin and soft tissue infections, particularly in neonates who present with localized erythema, induration, and systemic signs such as fever. In our case, the presence of hypercalcemia raised the index of suspicion for SCFN, but the eventual discovery of a deep abscess confirmed coexisting MRSA infection.

SCFN is primarily diagnosed clinically; however, biopsy remains the gold standard for definitive confirmation [1,2]. Ultrasound imaging can be a helpful adjunct, although sonographic findings can be difficult to distinguish from those of cellulitis or abscess, particularly in the initial stages [5,6,8]. SCFN typically appears as lobulated hyperechoic lesions within the subcutaneous fat layer, with well-defined margins [5]. In cases of hypercalcemia, associated calcifications may be visible and appear as acoustic shadows on ultrasound [1].

Differentiating SCFN from cellulitis can be especially challenging on ultrasound [5,8]. Both conditions may show increased echogenicity in the subcutaneous tissue [5,6,8]. However, a key distinguishing feature is the pattern of edema [6]. While cellulitis typically progresses with prominent, edematous hypoechoic striations that divide the subcutaneous fat into lobules, SCFN usually lacks such progressive edema [5,6,8]. Furthermore, SCFN, compared with cellulitis, lacks the increased blood flow observed on Doppler.

In comparison to abscesses, SCFN can be more readily distinguished sonographically when the abscess appears as a clearly defined hypoechoic fluid collection [6]. However, diagnostic confusion arises when an abscess appears hyperechoic or isoechoic relative to surrounding tissues. In such cases, clinical correlation is essential to reach an accurate diagnosis [1].

SCFN is generally a sterile condition, and the coexistence of infection is exceedingly rare [4,7]. In this case, we observed an unusual presentation of SCFN complicated by abscess formation. Ricardo-Gonzalez et al. reported on a neutrophil-rich SCFN lesion with expressible purulent fluid. They highlight that this SCFN variant was sterile but report that infection was a significant differential to consider [4].

Other differentials that may be challenging to distinguish from SCFN include cold panniculitis and sclerema neonatorum [1]. In both conditions, the lobulated fat pattern and nodules seen in SCFN are absent. Furthermore, subcutaneous tissue calcifications, a unique finding in SCFN, would not be observed in cold panniculitis or sclerema neonatorum [1,8]. Sclerema neonatorum also displays decreased compressibility due to subcutaneous and dermal hardening [8]. It is important to highlight that clinical correlation and lesion biopsies are the primary methods for diagnosing these lesions.

## Conclusions

The case presented in this report is one of the few reported cases in which SCFN coexisted with a bacterial abscess. The initial absence of purulent drainage and the delayed development of fluctuance contributed to the diagnostic uncertainty. This case emphasizes the importance of maintaining a broad differential diagnosis for neonatal soft tissue lesions and the need for serial imaging and specialist consultation when clinical evolution is atypical. In the context of rising serum calcium levels, it is imperative that SCFN be recognized in a timely manner. In the present case, the patient’s calcium levels remained elevated at discharge, necessitating ongoing outpatient monitoring to prevent complications such as nephrocalcinosis or cardiac arrhythmias.
